# Antimicrobial Resistance and Genomic Characterization of an *Escherichia coli* Strain Harboring p0111 and an IncX1-Type Plasmid, Isolated from the Brain of an Ostrich

**DOI:** 10.3390/vetsci12090793

**Published:** 2025-08-22

**Authors:** Jing Hu, Jiahe Zhou, Leping Wang, Zhongwei Chen, Yizhou Tan, Yangyan Yin, Zhe Pei, Changting Li, Huili Bai, Chunxia Ma, Ling Teng, Yongcui Feng, Xian Li, Yingyi Wei, Hao Peng

**Affiliations:** 1 College of Animal Science and Technology, Guangxi University, Nanning 530004, China; 2 Guangxi Key Laboratory of Veterinary Biotechnology, Guangxi Veterinary Research Institute, Nanning 530001, China; 3 Key Laboratory of China (Guangxi)-Association of Southeast Asian Nations (ASEAN) Cross-Border Animal Disease Prevention and Control, Ministry of Agriculture and Rural Affairs of China, Nanning 530001, China; 4 School of the Integrated Chinese and Western Medicine, Hunan University of Chinese Medicine, Changsha 410208, China; 5 School of Neuroscience, Virginia Tech, Blacksburg, VA 24061, USA; 6 Guangxi Agricultural Engineering Vocational Technical College, Nanning 532100, China

**Keywords:** *Escherichia coli*, ESBL, multidrug resistance, virulence factors, ostrich, whole-genome sequencing

## Abstract

An outbreak characterized by clinical signs of diarrhea and paralysis, occasionally progressing to fatal outcomes, occurred at an ostrich breeding facility. Conventional antibiotic treatments proved ineffective. To investigate the etiology of the disease, brain and liver specimens were collected for diagnostic analysis. An *Escherichia coli* (*E. coli*) isolate, designated strain HZDC01, was obtained from cerebral tissues, and whole-genome sequencing was performed for genomic characterization. Genomic analysis revealed that the chromosomal DNA harbors numerous resistance genes, conferring multidrug resistance through complex mechanisms. Furthermore, a p0111-type plasmid carrying the *bla_CTX-M-55_* gene and an IncX1-type plasmid harboring *rmtB*, *sul1*, *APH(6)-Id*, *tet(A)*, *AAC(3)-IIc*, *aadA2*, *bla_TEM-1B_*, and *floR* genes were identified. These plasmids carry numerous mobile genetic elements that can disseminate via horizontal gene transfer, thereby amplifying the risk of resistance-gene spread within bacterial populations. Additionally, the *ibeB* and *ibeC* genes, which encode proteins involved in the invasion of brain microvascular endothelial cells, were identified. These genes may facilitate *E. coli* penetration of the blood–brain barrier, potentially leading to meningitis and posing a life-threatening risk to the host. This is the first report of the isolation and characterization of extended-spectrum beta-lactamase *E. coli* from the brain of an ostrich with paralysis. The findings provide valuable genomic insights into the antimicrobial resistance profiles and pathogenic mechanisms of ostrich-derived *E. coli* isolates.

## Simple Summary

In an ostrich farm outbreak in Nanning, China, characterized by diarrhea and paralysis, a bacterial strain was isolated from the brain of a deceased ostrich. Whole-genome sequencing and bioinformatics analysis identified it as a multi-drug-resistant ESBL *Escherichia coli*. This strain carries type I fimbriae for adhesion and *ibe* family virulence factors that enable blood–brain barrier penetration. It exhibits resistance to β-lactams, quinolones, aminoglycosides, sulfonamides, and tetracyclines, thus complicating treatment. Additionally, plasmid-borne resistance genes co-localize with multiple mobile genetic elements capable of mediating their dissemination, posing a severe threat to both ostrich production and public health.

## 1. Introduction

*Escherichia coli (E. coli)* is a Gram-negative bacillus (GNB) widely present in the intestines of birds, typically existing as a symbiotic bacterium in the avian body. However, under certain conditions, such as stress, immunosuppression, or pathogen invasion, *E. coli* can transform into a pathogenic bacterium, leading to severe diseases including diarrhea, conjunctivitis, septicemia, toxemia, serositis, and salpingitis [1,2,3]. *E. coli* infections are one of the main causes of increased mortality in ostriches [4], resulting in significant economic losses to the poultry industry.

Recently, due to the inappropriate and irregular use of antimicrobial drugs, multidrug resistance (MDR) in *E. coli*, especially the production of extended-spectrum β-lactamases (ESBLs), has evolved into one of the most serious challenges in the global public health arena [5]. ESBLs are capable of hydrolyzing a variety of β-lactam antibiotics, including third-generation cephalosporins and monobactams, rendering these commonly used antimicrobials ineffective [6,7]. Among ESBLs, the *bla_CTX-M_* (ceftazidime-hydrolyzing) family has become the most prevalent, surpassing the *bla_TEM_* and *bla_SHV_* families [8]. The *bla_CTX-M_* gene is frequently associated with insertion sequences (ISs), transposable elements, and integrons, which facilitate its transfer and expression, thereby accelerating the horizontal spread of resistance genes [9]. Plasmids such as IncA/C, IncF, and IncX play a crucial role in the dissemination of *bla_CTX-M_* by transferring antimicrobial resistance genes (ARGs) between bacteria through conjugation [10]. Notably, phage-like plasmids (P-Ps) also contribute significantly to the spread of *bla_CTX-M_*. These plasmids combine the lysogenic and replicative capabilities of phages with the mobility of plasmids, allowing them to spread horizontally via phage particles and vertically within host cells [11], thus creating new pathways for the interspecies transmission of *bla_CTX-M_*.

Therefore, a comprehensive understanding and analysis of the ESBL-producing *E. coli* genome is vital for blocking ESBL transmission and devising better countermeasures. Recently, whole-genome sequencing (WGS) technology, combined with bioinformatics tools and databases, has proven to be a better choice [12]. It enables a deep analysis of the pathogenic mechanisms and transmission characteristics of isolated strains, offering key technical support for accurate traceability and real-time monitoring.

This study aims to comprehensively characterize an MDR *E. coli* strain isolated from an ostrich. Through systematic molecular analysis, we will determine its plasmid genotype, profile-associated resistance determinants (e.g., transposons and integrons), virulence factors, and potential immune evasion strategies. This integrated approach seeks to precisely delineate the mechanisms underpinning ARGs’ dissemination, assess virulence potential, understand host–pathogen interactions, and evaluate the potential boundaries of transmission.

## 2. Materials and Methods

### 2.1. Outbreak Description and Field Response

The ostrich farm had a flock size of approximately 320 ostriches. During this outbreak, a total of 187 ostriches exhibited symptoms, primarily characterized by paralysis and diarrhea. The affected ostriches showed signs of depression, anorexia, lethargy, and an inability to stand. In response to the outbreak, the farmer implemented the following measures: The entire farming environment was disinfected with a 2% sodium hydroxide solution (Zhongtai Chemical, Xinjiang, China). In accordance with the manufacturer’s instructions, injections of gentamicin sulfate (Quanyu Animal Pharmaceutical, Shanghai, China) and tetracycline hydrochloride (Xinercheng Animal Pharmaceutical, Jiangxi, China) were administered to the affected ostriches at doses corresponding to their body weights, twice daily for three consecutive days. Additionally, injections of florfenicol (Huachu, Henan, China) were given every 48 h for a total of two injections. After a course of treatment, the condition did not improve. Subsequently, the farmer added colistin sulfate (Bairuixiang, Zhengzhou, China) injections to the treatment, but ostriches were still dying, among which the chicks died within a week. To prevent the spread of the pathogen, the affected ostriches were culled and subjected to harmless treatment.

### 2.2. Specimen Collection and Viral Detection

To identify the causative pathogens, samples were collected from two randomly selected dead chick ostriches for viral detection via polymerase chain reaction (PCR). Intestinal, liver, and brain tissue samples were individually placed in 2 mL grinding tubes, homogenized with phosphate-buffered saline (PBS) (Servicebio, Wuhan, China), and subjected to three freeze-thaw cycles. Viral DNA/RNA was extracted using a commercial kit (CWBIO, Jiangsu, China) according to the manufacturer’s protocol. The resulting nucleic acids served as templates for detecting avian paramyxovirus-1 (APMV-1), avian encephalomyelitis virus (AEV), avian orthoreovirus (ARV), goose parvovirus (GPV), and mycoplasma synoviae (MS). Primer sequences were referenced from Zhao [13]. For RNA viruses, the extracted nucleic acids are first reverse-transcribed into cDNA using the gDNA Removal cDNA Synthesis Kit (CWBIO, Jiangsu, China), followed by detection. For each tissue, three samples were collected, and three parallel replicates were set up.

### 2.3. Identification of the Bacterial Strain

Under aseptic conditions, a scalpel, sterilized by burning (to inactivate potential contaminating bacteria with instantaneous high temperature), was gently touched to the surface of the tissue. Next, a sterile blade was used to incise the surface that had been touched by the instantaneous high temperature. Subsequently, the sterilized inoculation loop was inserted into the tissue through the incision to obtain an appropriate amount of tissue, which was then streaked onto the Luria–Bertani (LB) agar plates and MacConkey agar (MAC) plates (Land Bridge, Beijing, China). All plates were inverted and incubated at 37 °C for 24 h. Characteristic colonies were then selected based on morphological features and transferred to appropriate culture media for purification.

### 2.4. 16S rRNA Sequencing

The 16S rRNA gene was amplified by PCR using universal primers 27F (5′-AGAGTTTGATCCTGGCTCAG-3′) and 1492R (5′-TACGGCTACCTTGTTACGACTT-3′). The 25 μL PCR mixture contained 12.5 μL 2× Es TaqMasterMix (Dye) (CWBIO, Jiangsu, China), 1 μL of each primer, 2 μL of template, and 8.5 μL of ddH_2_O. Amplification conditions were denaturation at 95 °C for 10 min, followed by 35 cycles of denaturation at 95 °C for 50 s, annealing at 52 °C for 40 s, and elongation at 72 °C for 1 min, followed by a final extension at 72 °C for 5 min. The 16S rDNA amplicon was sequenced by a commercial sequencing facility (Sangon, Shanghai, China). For each isolate, three individual colonies were collected, and three parallel replicates were set up for sequencing.

### 2.5. Whole-Genome Sequencing

Genomic DNA was extracted using the Bacterial Genomic DNA Kit (CWBIO, Jiangsu, China) according to the manufacturer’s instructions, and the total amount of DNA was evaluated by using the Quant-iT PicoGreen dsDNA Assay Kit (Thermo Fisher Scientific, Waltham, USA). The library was prepared using TruSeqTM DNA Sample Prep Kit with a library insert size of 400 bp, and 150 bp paired-end sequencing was performed using the Illumina NovaSeq platform. Furthermore, third-generation sequencing (TGS) of genomic DNA was performed by using the Oxford Nanopore (ONT) platform implemented in accordance with standard protocol. AdapterRemoval v2.2.2 [14] software was employed to trim NovaSeq reads, while SOAPec v2.03 [15] was utilized for quality correction of all reads based on Kmer (length 17) frequency. TGS data were assembled using HGAP v4 [16] and CANU v1.7.1 [17] software to generate the contig sequence. Thereby, the contig sequences were corrected by high-quality data of Illumina sequencing via Pilon v1.18 [18] software and finally spliced to obtain the complete sequence.

### 2.6. Bioinformatics Analysis

The assembled sequences were submitted to KmerFinder 3.2, PlasmidFinder 2.1, pMLST 2.0, and SerotypeFinder 2.0 to evaluate the bacterial species, plasmid types, multi-locus sequence typing, serotype, and phylogenetic group of the isolate. The tools mentioned above are available on the website of the Center for Genomic Epidemiology (CGE, https://www.genomicepidemiology.org/services/, accessed on 5 July 2025). Phylogroup assignation was performed using ClermonTyping v23.06 (http://clermontyping.iame-research.center/, accessed on 25 June 2025) [19]. The ARGs were detected using Comprehensive Antibiotic Resistance Database v4.0.1 (CARD, https://card.mcmaster.ca/, accessed on 2 June 2025) [20]. VFanalyzer v4.0 was used to determine the virulence factors (VFs) (http://www.mgc.ac.cn/VFs/main.htm, accessed on 8 June 2025) [21]. High-quality complete genome sequences were annotated using Rapid Annotation using Subsystem Technology v (RAST, https://rast.nmpdr.org/, accessed on 14 June 2025) [22] and curated manually using the BLASTn and BLASTp algorithms (https://blast.ncbi.nlm.nih.gov/Blast.cgi, accessed on 15 June 2025). Characterization and visualization of chromosomes and plasmids were generated using Proksee (https://proksee.ca/, accessed on 8 July 2025) [23]. Gene ontology (GO) annotation of protein-coding genes was carried out using BLAST2GO v6.0 (https://www.blast2go.com/, accessed on 4 July 2025) [24]. Easyfig 2.2.5 is creating linear comparison figures of genomic loci based on BLAST [25]. The similarity comparison of the genomes of the isolated strains was conducted using the ANI Calculator (https://www.ezbiocloud.net/tools/ani, accessed on 20 May 2025) [26]. KO annotations were performed using KAAS [27] automated annotation system of Kyoto Encyclopedia of Genes and Genomes v115.0 (KEGG, https://www.genome.jp/kegg/, accessed on 4 July 2025).

### 2.7. Antibiotic Susceptibility Testing (AST)

According to the Clinical Laboratory Standard Institute guidelines (CLSI) [28], the antibacterial susceptibility testing of the HZDC01 strain was conducted using the Kirby–Bauer (K-B) disk diffusion method. The strain was tested for susceptibility against a range of 23 antimicrobial discs (Hangzhou microbial reagent, Hangzhou, China), including spectinomycin (SPC: 100 μg), sulfamethoxazole/trimethoprim (SXT: 25 μg), kanamycin (K: 30 μg), enrofloxacin (ENR: 5 μg), amoxicillin (AML: 10 μg), ofloxacin (OFX: 15 μg), gentamicin (GEN: 10 μg), ampicillin (AMP: 10 μg), ciprofloxacin (CIP: 5 μg), cefotaxime (CTX: 30 μg), cefoxintin (FOX: 30 μg), doxycycline (DOX: 30 μg), tetracycline (TET: 30 μg), ceftriaxone (CRO: 30 μg), ceftazidime (CAZ: 30 μg), tobramycin (TOB: 10 μg), meropenem (MEM: 10 μg), imipenem (IPM: 10 μg), amikacin (AMK: 30 μg), norfloxacin (NOR: 10 μg), florfenicol (FFC: 30 μg), polymyxin B (PB: 30 μg), and azithromycin (AZM: 15 μg). The quality control organism was *E. coli* ATCC 25922. After placing the antibiotic discs on the inoculated agar plates, they were incubated at 37 °C for 24 h. The zones of inhibition around each disc were measured to determine the susceptibility of the strain. The interpretive criteria used were those recommended for Enterobacteriaceae according to the CLSI standards.

### 2.8. Nucleotide Sequence Accession Numbers

The complete genome of the HZDC01 isolate was deposited in GenBank under the accession numbers CP118681.1 (chromosome), CP118682.1 (plasmid 1), CP118683.1 (plasmid 2), and CP118684.1 (plasmid 3).

## 3. Results

### 3.1. Tissue and Pathological Assessment with Bacterial Strain Identification

**Figure 1:**
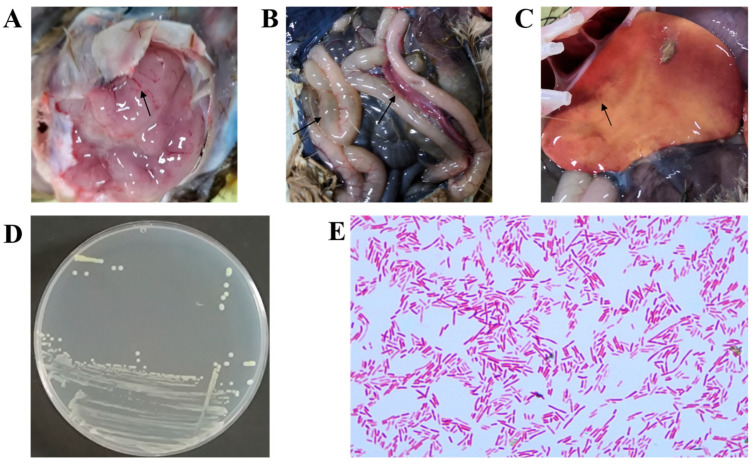
Histopathologic findings in deceased ostriches. (**A**) The brain exhibited cerebral edema associated with mild cerebral hyperemia. (**B**) The pancreas was congested, and the intestinal walls were notably thinned. (**C**) The liver showed yellowing and petechial hemorrhages. (**D**) Round, smooth, moist, and milky-white translucent colonies were cultured on an LB agar plate from a brain sample. (**E**) GNB were observed in the brain tissue.

Necropsy of two afflicted ostriches revealed consistent tissue lesions. As depicted in Figure 1, the brain showed signs of edema and mild congestion (Figure 1A). The pancreas appeared congested, and the intestinal walls were notably thinned (Figure 1B). The liver was enlarged, displaying a yellowish coloration and punctate hemorrhages (Figure 1C).

Based on the clinical presentation of diarrhea and paralysis in the affected ostriches, viral isolation was performed as the initial diagnostic approach. APMV-1, AEV, ARV, GPV, and MS were detected via PCR. Testing revealed that none of these pathogens were detected (Figures S1 and S2).

To identify the causative pathogen, brain and liver samples from two deceased ostrich chicks were collected for biological examination. The brain tissue samples were particularly crucial, as the isolated strains formed distinctive pink to red colonies on MAC agar. After purification on LB agar, these colonies exhibited characteristics of being round, raised, smooth, moist, and creamy white (Figure 1D). Gram staining revealed that the isolate was a GNB (red short rods, Figure 1E). 16S rRNA sequencing identified the strains isolated from the brains of the two deceased ostrich chicks as *E. coli.* Further WGS analysis revealed that the two brain-derived strains had a high degree of homology (ANI = 99.98%), indicating that they were the same clone. This finding suggests that *E. coli* might be the pathogen responsible for the outbreak.

KmerFinder indicated that the closest genome to this strain is *E. coli* strain C21 (NZ_CP052877.1) with 97.81%. The analysis indicated that the strain belonged to the B1 phylogenetic group and sequence type (ST) 156, which has been widely found among humans, poultry, and livestock all over the world (ST-156 entry from EnteroBase). Serotype was determined by the genotype of fliC, wzx, and wzy, and the results showed that the strain belonged to O51:H28. Consequently, the isolate was identified as *E. coli* and named HZDC01.

### 3.2. General Information About the Chromosomal Genome of the HZDC01 Strain

**Figure 2:**
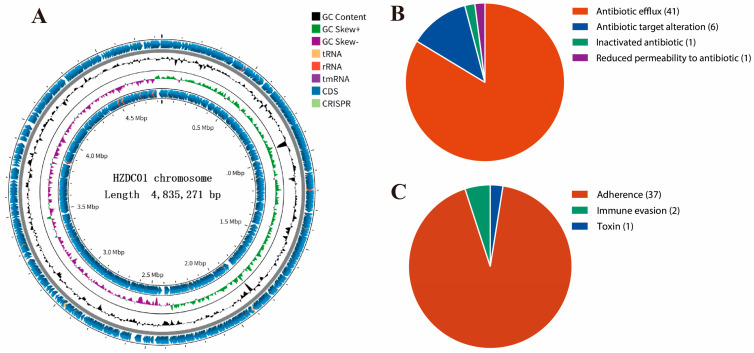
Detailed visual insights into the chromosomal genome of the HZDC01 strain. (**A**) A comprehensive summary of gene annotation and GC skew analysis of the HZDC01 genome. The depiction consists of several concentric circles: Circle 1 represents the scale of the genome. Circles 2 and 5 indicate the positions of coding sequences (CDSs) within the genome. Circle 3 displays the GC skew, which helps in identifying the origin and termination of replication in the genome. Circle 4 shows the overall GC content, providing insight into the genomic composition. (**B**) Classification of ARGs in the chromosomal genome categorizes the different types of ARGs found within the chromosome, highlighting genes associated with antibiotic efflux, target alteration, inactivation, and reduced permeability. (**C**) Classification of VFs in the chromosomal genome classifies the VFs identified in the genome analysis, grouping them by their functions, such as adherence, immune evasion, and toxin production.

To achieve a comprehensive understanding of the genetic makeup of the HZDC01 strain, it underwent WGS using the Illumina NovaSeq platform and third-generation sequencing technologies. The sequencing process achieved a coverage of 206×, producing 6,851,204 high-quality reads, with 99.02% of them classified as high-quality, accurately reflecting the actual nucleotide composition of the strain. A total sequence length of 1,029,659,932 bp and a GC content of 50.8% were obtained on the ONT platform. The complete genome of the HZDC01 strain comprises one circular chromosome and three circular plasmids. The chromosome itself is 4,835,271 bp in size, with a GC content of 50.71%, and contains 4505 open reading frames (ORFs), 22 rRNA genes (including 8 5S rRNAs, 7 16S rRNAs, and 7 23S rRNAs), and 86 tRNA genes (Figure 2A). The analysis of ARGs in the chromosome revealed diverse mechanisms of resistance (Figure 2B). Specifically, the prediction results identified 41 genes associated with antibiotic efflux, six genes linked to alterations in antibiotic targets, one gene related to antibiotic inactivation, and one gene contributing to reduced antibiotic permeability (Table S1). Additionally, 40 VFs were detected, including 37 factors related to adherence, two associated with immune evasion, and one toxin (Figure 2C).

### 3.3. Detection of Resistance Genes

ARGs in the HZDC01 strain have been identified, located on the chromosome and on plasmids. Specifically, a multitude of ARGs within the chromosome encompass a diverse array of resistance mechanisms. The prediction results identified 41 genes associated with antibiotic efflux, one gene contributing to reduced antibiotic permeability, six genes linked to alterations in antibiotic targets, and one gene related to antibiotic inactivation (Table 1). Notably, the identification of multiple mutated resistance genes, such as *marR*, *acrR*, *soxS*, and *soxR*, may be associated with the exacerbation of multidrug resistance.

**Figure 3:**
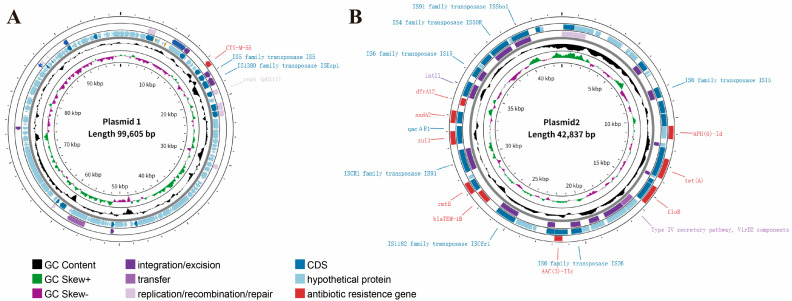
Characterization and visualization of the plasmid genome. (**A**) Characterization of HZDC01 P1 genome. (**B**) Characterization of HZDC01 P2 genome. The depiction consists of several concentric circles: Circle 1 represents the scale of the genome. Circle 2 represents GC skew. Circle 3 represents GC content. Circles 4 and 5 indicate CDS and bacterial mobile genetic elements annotation, including integration/excision, transfer, and replication/recombination/repair. Circle 6 represents ARGs’ prediction at the CARD.

Within the genome of HZDC01, three plasmids were identified: plasmid 1 (P1) with the p0111 replicon, plasmid 2 (P2) with the IncX1 replicon, and plasmid 3 with no known replicon (Table 2 and Figure 3). ARGs located on mobile genetic elements (such as plasmids, integrons, and insertion sequences) are highly transferable between bacterial species [29]. Plasmid 1 harbors *bla_CTX-M-55_*, endowing the strain with resistance to third-generation cephalosporins. P1 showed high nucleotide identity (99.97%) with phage JL22 (GenBank accession ON018986.2), differing only by a few SNPs in non-coding regions. Its core functional modules, including the p0111 replication protein gene, IScp1 and IS5, and the *bla_CTX-M-55_* gene, were found to be highly conserved during evolution (Figure 3A and Figure S3). Plasmid 2 carries *rmtB*, *sul1*, *APH(6)-Id*, *tet(A)*, *AAC(3)-IIc*, *aadA2*, *bla_TEM-1B_*, *floR*, conferring resistance to aminoglycosides, sulfonamides, penicillins, tetracyclines, and chloramphenicol. And multiple insertion sequences (ISs) were identified in P2, such as IS15, IS91, IS50R, and ISCfrr1, and a class 1 integron (*intI1*) was discovered. Furthermore, only VirD 2, a component of the Type IV secretory pathway, was detected, whereas the essential channel proteins of the T4SS (e.g., VirB4, VirB11, and VirD4) are absent (Figure 3B); plasmid 3 with no ARGs.

### 3.4. Virulence Factors of the HZDC01 Strain

To elucidate the virulence mechanisms of the HZDC01 strain, we conducted a comprehensive analysis and identified multiple VFs involved in various mechanisms (Table 3). Hemolysin E (*hlyE/clyA*) is a novel agaricus bisporus protein found in *E. coli*, *Salmonella typhimurium*, and *Shigella flexneri* [30]. It is known to mediate monocytes and macrophages’ lysis [31]. It has been reported that *hlyE* is involved in the pathogenic mechanism of *E. coli*, and its production leads to extraintestinal infections [32]. Additionally, the *ibe* genes, including *ibeB* and *ibeC*, which are associated with the invasion of brain endothelial cells, were identified. These genes are part of the *ibe* family and are critical in enabling *E. coli* to penetrate brain microvascular endothelial cells (BMECs) in vitro and to permeate the blood–brain barrier (BBB) in vivo [33]. Other significant VFs identified include type I fimbriae and *eaeH*, along with factors belonging to the *esp* family, particularly *espX5*. Type I fimbriae are known to mediate specific binding between bacteria and eukaryotic cells. The *eaeH* gene encodes a variant of an inner-membrane protein that may facilitate epithelial attachment and subsequent lesion effacement [34]. *espX5* is located on the locus of enterocyte effacement (LEE), which encodes for the type III secretion system of enteropathogenic *E. coli* (EPEC) [35]. This locus primarily codes for proteins involved in intimate adherence to the intestinal mucosa, a crucial step in pathogenesis.

### 3.5. HZDC01 Exhibits Multidrug Resistance

In the study, antibiotic susceptibility testing was conducted on HZDC01 using 23 different antibiotics (Table 4). HZDC01 demonstrated resistance to a range of antibiotics commonly used in medical treatments, food animal feed, and veterinary medicine. However, the strain showed sensitivity to cefoxitin and imipenem and intermediate sensitivity to doxycycline. In addition, the inhibition zone diameter for the polymyxin B disk diffusion reached 15 mm. This susceptibility profile reveals that the strain is susceptible to major antibiotic classes, including aminoglycosides, β-lactams, quinolones, sulfonamides, and tetracyclines. This resistance pattern underscores the challenge posed by HZDC01, particularly in contexts where these antibiotics are routinely used, and highlights the need for ongoing surveillance and judicious use of antibiotics to manage the risk associated with such resistant strains.

### 3.6. Functional Annotation of Protein-Coding Genes of HZDC01

**Figure 4:**
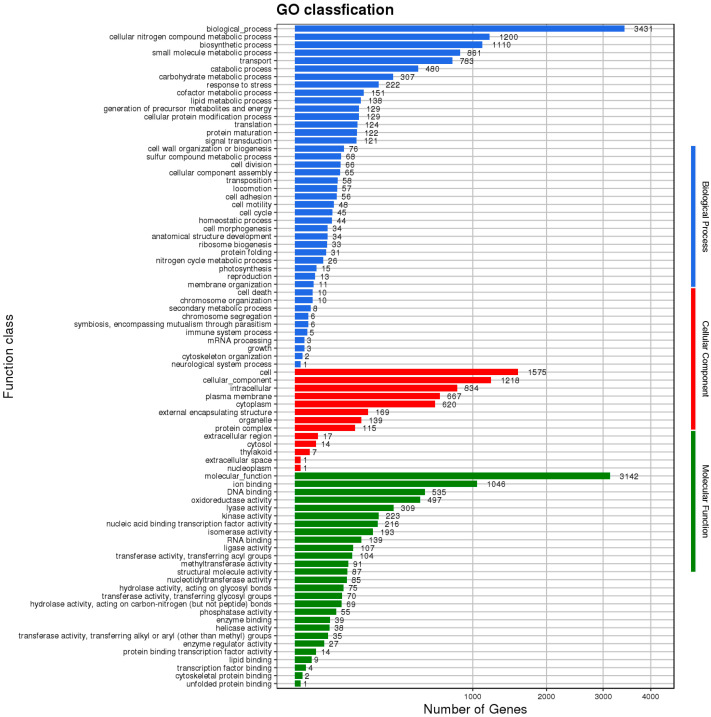
The Gene Ontology (GO) functional classification diagram of HZDC01. The result was displayed by using map2slim.

The genomic sequence of HZDC01 was comprehensively annotated, as depicted in Figure 4. A total of 3431 coding sequences were annotated, assigned to 83 distinct Clusters of Orthologous Groups (COG) categories. These categories were further divided into three primary functional groups. The Biological Process group accounted for 51.81% of the annotated sequences, reflecting their direct involvement in dynamic biological activities. The Cellular Component group represented 15.66% of the annotations, indicating their essential roles in cellular architecture and intracellular processes. The Molecular Function group comprised 32.53% of the annotated genes, representing molecular-level activities within the cell.

**Figure 5:**
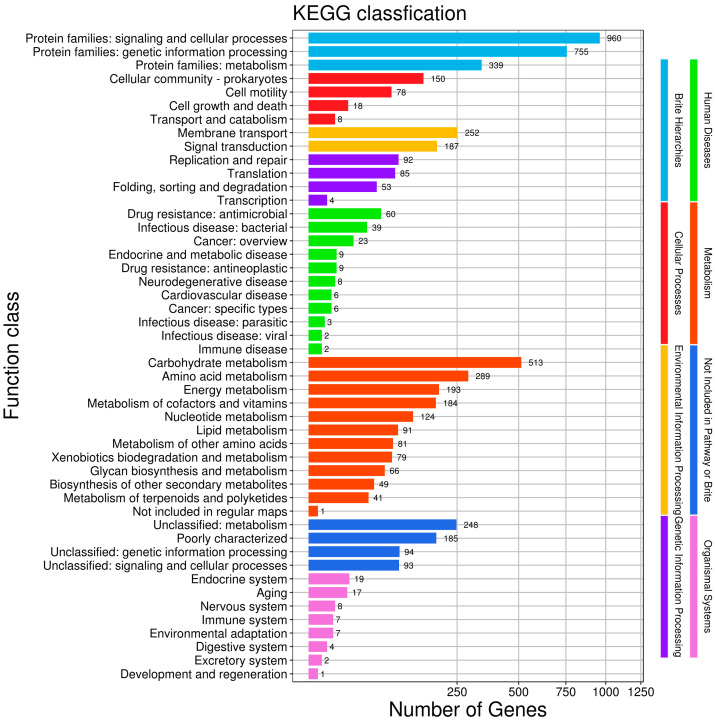
The Kyoto Encyclopedia of Genes and Genomes (KEGG) function annotation of HZDC01. The gene set was selected as “For Prokaryotes”, and bi-directional best hit (BBH) was selected as the discriminant rule for KO.

To enhance our understanding of gene functions in HZDC01, a comprehensive analysis was conducted by mapping 4924 putative proteins to their orthologs in the KEGG database, as illustrated in Figure 5. These proteins were categorized into six main functional groups: (1) Cellular Processes: 254 proteins, focusing on fundamental cellular mechanisms. (2) Environmental Information Processing: 439 proteins, involved in the processing of environmental signals and responses. (3) Genetic Information Processing: 234 proteins, dealing with the mechanisms of genetics such as replication, transcription, and translation. (4) Human Diseases: 167 proteins, linked directly to pathogenicity and interactions with human hosts. (5) Metabolism: 1711 proteins, encompassing a broad range of metabolic pathways that support bacterial survival and growth. (6) Organismal Systems: 65 proteins, which play roles in the system-level operations of the organism.

## 4. Discussion

*E. coli* occupies a dual role as both a commensal microorganism and an opportunistic pathogen, with its infections potentially leading to lethal systemic infections. In recent years, the evolution of MDR in *E. coli* has emerged as a core threat, significantly diminishing the clinical efficacy of first-line antimicrobial agents. This resistance expansion is essentially driven by mobile genetic elements, which induce adaptive genetic variations that subsequently influence treatment decisions through predictable phenotypic expressions. Therefore, investigating the correlation between resistance genotype and phenotype, as well as the synergistic mechanisms of mobile genetic elements in the isolated strain HZDC01, will provide key targets for elucidating the evolutionary pathways of MDR and devising precise intervention strategies.

Genotypic and phenotypic analyses of the HZDC01 demonstrated concordant resistance profiles to β-lactams, quinolones, aminoglycosides, sulfonamides, and tetracyclines. Notably, this strain harbors a significant number of genes encoding efflux pumps, which are bacterial inner membrane transporters capable of expelling a variety of molecules, such as toxins and antimicrobials, from the bacterial cell [36]. Efflux pumps are generally chromosomally located and highly conserved. Nevertheless, in the case of continuous exposure to antibiotics, certain genes can acquire mutations. These random mutations may lead to the overexpression of efflux pumps, which in turn can result in multidrug resistance [37,38]. *soxS* functions as a transcription factor that is activated by *soxR* under oxidative stress conditions [39]. The activation of the SoxR-SoxS system leads to the upregulation of multidrug efflux pump genes, thereby facilitating the expulsion of antimicrobial agents [40]. Furthermore, research has shown that mutations in the *soxR* gene can result in elevated SoxS expression levels, which in turn assist the strain in acquiring multidrug resistance [41]. In addition to these findings, *soxS* upregulates the expression of the AcrAB efflux pump gene *acrB* [40] and simultaneously reduces the expression of OmpF to decrease membrane permeability [42]. This elucidates the mechanism underlying the resistance of HZDC01 to meropenem while retaining sensitivity to imipenem. Specifically, the overexpression of the AcrAB-TolC efflux system is one of the key factors contributing to meropenem resistance [43]. Moreover, the decreased expression of porin OmpF exacerbates the challenge for the more hydrophilic meropenem to penetrate the cell membrane [44]. Additionally, *acrR* acts as a repressor of the AcrAB-TolC multidrug efflux complex, and mutations in *acrR* can lead to high levels of antibiotic resistance [45]. *marR* is a repressor of the mar operon marRAB, and its mutations can modulate the expression of MarA, an activator of the multidrug efflux pump AcrAB-TolC [46,47]. Mutations in *marR* and *acrR* can confer resistance to ciprofloxacin, tetracycline, and ceftazidime. Fluoroquinolones inhibit DNA replication by targeting DNA gyrase (topoisomerase II) and topoisomerase IV [48]. In our study, resistance to fluoroquinolones in the HZDC01 strain was primarily attributed to mutations in the fluoroquinolone resistance-determining regions (FRDRs) of the *gyrA* and *parC* genes, which encode subunits of DNA gyrase and topoisomerase IV, respectively [49,50]. These mutations reduce the binding affinity of fluoroquinolones to their target enzymes, thereby conferring resistance.

In clinical settings, ST156 *E. coli* strains frequently exhibit the *mcr-1* gene conferring colistin resistance along with carbapenem resistance genes (such as *bla_KPC_*, *bla_NDM_*, *bla_VIM_*) [51,52,53]. Carbapenem antibiotics are recognized as last-resort agents for treating MDR GNB infections, while colistin has emerged as the ultimate line of defense against carbapenem-resistant GNB strains [54,55]. Furthermore, VF profiling indicates that *mcr*-*1* frequently coexists with *terC* and *gad* virulence-associated genes [56]. However, it is noteworthy that the mcr-1 gene was not detected in either the chromosome or the plasmids of HZDC01. Correspondingly, the aforementioned VFs were also undetected. These findings indicate that the strain may be susceptible to colistin. According to the AST result, polymyxin B exhibited an inhibition zone (15 mm) against HZDC01. Although CLSI explicitly states that the disk diffusion method is not suitable for polymyxin susceptibility testing [57], the initial results, in combination with the genotypic findings, suggest that HZDC01 has intermediate resistance to colistin. This may account for why HZDC01 was isolated from brain tissue but not from liver samples. Colistin sulfate, while able to inhibit HZDC01, cannot cross the BBB and is ineffective against bacteria in the brain tissue. Also, although gentamicin can cross the BBB, it is ineffective against the drug-resistant HZDC01 strain. The failure to isolate HZDC01 from liver samples might be because colistin sulfate cleared the pathogenic bacteria in the liver.

In the HZDC01 P1, a typical seat unit structure is present: ISEcp1-*bla_CTX-M-55_*-orf477. The ISEcp1 upstream of *bla_CTX-M-55_* has been confirmed to promote the expression of downstream genes [58], mediate the transposition of ARGs from the chromosome to the plasmid [59], and is considered one of the key mechanisms driving the rapid spread of *bla_CTX-M-55_*. The study by Wang demonstrated that JL22, as a p-p1-like plasmid, successfully transferred *bla_CTX-M-55_* to naturally isolated *E. coli* MG1655 via lysogenization [60]. This also suggests that HZDC01 p1 belongs to the p-p1-like plasmid, which facilitates the spread of *bla_CTX-M-55_* to other *E. coli* strains. HZDC01 was classified as sequence type ST156, which has been identified with multiple conjugative plasmids. For instance, the *E. coli* strain GZEC065 isolated in China was shown to harbor an IncX3 conjugative plasmid that facilitates the dissemination of *bla_NDM-5_* [53]. In the present study, we identified a conjugative plasmid, IncX1. IncX1 plasmids possess broad host-range compatibility within the Enterobacteriaceae and disseminate efficiently by conjugation across strains and even across species, with transfer frequencies reaching 10^−4^ [61]. The IS family represents the simplest mobile genetic elements known to modulate the expression of adjacent genes [62]. In plasmid p2, multiple IS, representing the IS4, IS6, IS91, IS1595, and ISCR1 families, were identified. The IS family represents the simplest class of mobile genetic elements, modulating the expression of adjacent genes [63].

Notably, the P2 harbors the ISCR1–sul1–qacΔE1–aadA2–dfrA12–intI1–IS6 resistance array, which integrates integron, transposon, and insertion-sequence elements. ISCR1, a member of the IS91 family, mobilizes adjacent DNA via rolling-circle replication, while its outward-oriented promoter (*Pout*) markedly up-regulates downstream antibiotic-resistance genes (e.g., *bla_CTX-M_* and *dfrA19*), thereby facilitating their horizontal dissemination [64]. The intI1 is frequently located adjacent to ISCR1 and typically carries sul1 and qaΔE1 within its 3′-conserved segment, conferring sulfonamide resistance and reduced susceptibility to quaternary ammonium disinfectants, respectively [63]. IS6 is recognized as one of the insertion-sequence families most intimately linked to the horizontal dissemination of antibiotic-resistance genes, being widely distributed among non-mobilizable, mobilizable, and conjugative plasmids. Via transposase-mediated transposition, IS6 can relocate itself together with adjacent resistance determinants, such as optrA, into novel plasmid or chromosomal loci [65]. Therefore, the “gene cassette enclosure” structure formed by ISCR1 and IS6 may mediate the precise excision and transfer of the central resistance module (from sul1 to intI1), enabling its integration into other plasmids, chromosomes, or bacteriophages. The widespread dissemination of this structure poses a severe environmental threat: Due to the co-localization of resistance genes against clinical antimicrobials, veterinary antibiotics, and environmental disinfectants, this plasmid acquires a persistent transmission advantage across multiple settings.

Apart from ARGs, identifying the pathogenicity of strains isolated from diseased animals is crucial. The identification of specific VFs might explain the clinical symptoms observed. Affected ostriches primarily manifested sublethal symptoms, including lethargy, ruffled feathers, diarrhea, and paralysis, with only a minority of chicks developing fatal outcomes; this phenotypic profile demonstrated consistency with VFs’ analysis results. For instance, Type I fimbriae and *espX5* facilitate the adhesion of bacteria to the surface of intestinal epithelial cells and the release of toxins, leading to diarrhea. Additionally, the paralysis observed in sick ostriches drew our attention. Various conditions, such as botulism [66] and parvovirus [13,67] infection, have been proposed to explain such symptoms, but these pathogens were not detected in the ostrich samples in this study. Notably, the genes *ibeB* and *ibeC* were discovered. Research by Huang indicated that *ibeB* is involved in the colonization and invasion of the host brain by avian pathogenic *E. coli* (APEC) [68]. Wang’s research elucidated the process by which *E. coli* causes meningitis: After entering the gastrointestinal tract mucosa, *E. coli* invades the intravascular space, traverses the BBB, and ultimately triggers diseases in the central nervous system [69]. Given that the strain was isolated from the brain of ostriches, we speculated that HZDC01 could enter the brain by crossing the BBB, causing cerebral edema, which resulted in paralysis and ultimately posed a life-threatening risk to the host. This hypothesis requires further validation through animal experiments, utilizing techniques such as bioluminescent imaging and real-time multimodality imaging [70].

When conducting an in-depth analysis of the pathogenic characteristics of this strain, the property enabling evasion of the host immune system’s killing mechanisms also calls for investigation. Cationic antimicrobial peptides (CAMPs) are crucial in the innate immune defense against bacterial infections [71]. HZDC01 has evolved various mechanisms to evade these peptides, notably through transport proteins. One significant discovery in the HZDC01 genome was the Sap transporter system, which includes *sapA*, *sapB*, *sapC*, *sapD*, and *sapF*. This system is critically important for antimicrobial peptide resistance in GNB. For example, the SapA protein in *Actinobacillus pleuropneumoniae* has been shown to confer defense against linear porcine antimicrobial peptides [72]. Additionally, the phoP/phoQ two-component system (TCS) was identified. This vital bacterial signal transduction system, comprising the histidine kinase phoQ and the response regulator *phoP*, mediates bacterial responses to external stimulation [73]. TCS is known to influence bacterial growth, biofilm formation, and virulence by regulating gene cluster expression [74,75]. The impact of TCS on pathogenicity was demonstrated in APEC by observing mutants with deletions in phoP or phoQ [76]. For instance, deletion of phoP in APEC strains inhibited biofilm formation and increased sensitivity to antibiotics [77], highlighting the system’s role in environmental adaptability and drug resistance.

In our study, HZDC01 was found to be resistant to imipenem and cefoxitin, while showing intermediate susceptibility to polymyxin B and doxycycline. Given the potential of HZDC01 to cross the blood–brain barrier, we reviewed relevant literature. The literature indicates that doxycycline, due to its good lipid solubility, can penetrate the BBB [78]. Additionally, although cefoxitin has difficulty crossing the BBB under normal physiological conditions, it can enter the cerebrospinal fluid and reach therapeutic concentrations in the presence of meningitis [79]. Therefore, both doxycycline and cefoxitin can be considered as potential treatment options for HZDC01 infections. Meanwhile, considering the early control of infection, polymyxin and imipenem can also be part of the treatment regimen.

While there are still options for the antibiotic treatment of HZDC01 infections, it is important to consider that under selective antibiotic pressure, the pathogen may evolve multiple resistance mechanisms and continuously develop strategies for the dissemination of resistance genes. Consequently, concepts emphasizing antibiotic reduction, substitution, or complete avoidance are increasingly being adopted as sustainable practices in China’s livestock industry [80]. In recent years, research efforts have shifted toward developing antibiotic alternatives, including probiotics [81], antimicrobial peptides [82], plant-derived extracts [83], and phage therapy [84]. Moving forward, identifying effective candidates for clinical application against MDR *E. coli* infections remains crucial. Furthermore, enhancing recognition of ARGs as a food safety threat is imperative. From a One Health perspective, strengthening surveillance of food-borne AMR bacteria to limit their entry into the food chain is essential for safeguarding public health and containing the spread of resistant infections.

To our knowledge, this is the first report of MDR *E. coli* isolated from ostriches characterized by paralysis. The results indicate that HZDC01 not only exhibits a multidrug-resistant phenotype but also harbors the potential to breach the BBB. By thoroughly elucidating the pathogenic mechanisms of HZDC01 and its adaptive strategies in hosts and the environment, this study provides critical evidence for its potential zoonotic transmission risk. These findings not only lay the groundwork for evidence-based risk assessment frameworks but also, from a One Health perspective, serve as a warning: Pathogens with high drug resistance and the ability to cross key physiological barriers pose a significant threat to human, animal, and environmental health if spread, constituting a triple challenge to health.

## Figures and Tables

**Table 1:** Antibiotic resistance genes in the HZDC01 chromosome.

Resistance Mechanism	AMR Genes Family	Antibiotic Resistance Genes
Antibiotic efflux	Major facilitator superfamily (MFS) antibiotic efflux pump	*emrA*, *emrB*, *emrR*, *emrK*, *emrY*, *mdtH*, *mdtM*, *mdtN*, *mdtO*, *mdtP*, *mdtG*, *leuO*, *mdfA*
	Small multidrug resistance (SMR) antibiotic efflux pump	*emrE*
	Resistance–nodulation–cell division (RND) antibiotic efflux pump	*acrA*, *acrB*, *acrD*, *acrE*, *acrF*, *acrS*, *mdtA*, *mdtB*, *mdtC*, *mdtE*, *mdtF*, *marA*, *cpxA*, *baeR*, *gadX*, *CRP*, *Ecol_marR_MULT*, *Ecol_acrR_MULT*,
	ATP-binding cassette (ABC) antibiotic efflux pump	*msbA*, *Yojl*,
	Major facilitator superfamily (MFS) antibiotic efflux pump, resistance–nodulation–cell division (RND) antibiotic efflux pump	*evgA*, *evgS*, *H-NS*
	ATP-binding cassette (ABC) antibiotic efflux pump, major facilitator superfamily (MFS) antibiotic efflux pump, resistance–nodulation–cell division (RND) antibiotic efflux pump	*tolC*, *Ecol_soxR_MULT*, *Ecol_soxS_MULT*
	kdpDE	*kdpE*
Reduced permeability to antibiotic		*marA*
Antibiotic target alteration	Elfamycin-resistant EF-Tu	*Ecol_EFTu_PLV*
	Fluoroquinolone-resistant gyrA	*Ecol_gyrA_FLO*
	Fluoroquinolone-resistant parC	*Ecol_parC_FLO*
	PMR phosphoethanolamine transferase	*pmrF*, *ugd*, *eptA*
	Undecaprenyl pyrophosphate-related proteins	*bacA*
Inactivated antibiotic	EC beta-lactamase	*EC-18*

**Table 2:** Information about the plasmid genome of the HZDC01 strain.

Itemize	Plasmid 1	Plasmid 2	Plasmid 3
Size (bp)	99,605	42,837	4018
GC content (%)	47.79	53.08	53.29
ORFs	109	51	4
Replicon	p0111	IncX1	None
ARGs	*bla* *_CTX-M-55_*	*rmtB*, *sul1*, *APH(6)-Id*, *tet(A)*, *AAC(3)-IIc*, *aadA2*, *bla_TEM-1B_*, *floR*	None
VFs	none	none	None

Note: To fully assess the resistance genes in HZDC01 plasmids, we employed a combination of tools and methods. We first used the CARD for resistance gene prediction, then validated and supplemented these results with RAST. Finally, we manually corrected the predictions using BLASTn (with ≥90% identity, ≥80% coverage, and E-value ≤ 1 × 10^−10^) to ensure accuracy.

**Table 3:** Virulence factors prediction of the HZDC01 strain.

Itemize	Virulence Factors	Related Genes
Adherence	CFA/I fimbriae	*cfaB*, *cfaC*, *cfaD/cfaE*
	*E. coli* common pilus (ECP)	*ecpA*, *ecpB*, *ecpC*, *ecpD*, *ecpE*, *ecpR*
	*E. coli* laminin-binding fimbriae (ELF)	*elfA*, *elfC*, *elfD*, *elfG*
	EaeH	*eaeH*
	Hemorrhagic *E. coli* pilus (HCP)	*hcpA*, *hcpB*, *hcpC*
	Type I fimbriae	*fimA*, *fimB*, *fimC*, *fimD*, *fimE*, *fimF*, *fimG*, *fimH*, *fimI*
	Cah, AIDA-I type	*cah*
Autotransporter	EhaB, AIDA-I type	*ehaB*
	UpaG adhesin, trimeric AT	*upaG/ehaG*
Invasion	Invasion of brain endothelial cells (Ibes)	*ibeB*, *ibeC*
Non-LEE encoded TTSS effectors	EspL1	*espL1*
	EspL4	*espL4*
	EspR1	*espR1*
	EspX1	*espX1*
	EspX4	*espX4*
	EspX5	*espX5*
Secretion system	ACE T6SS	*aec16*, *ace17*, *aec18*, *aec19*, *aec22*, *aec23*, *aec24*, *aec25*, *aec26*, *aec27/clpV*, *aec28*, *aec29*, *aec30*, *aec31*, *aec32*, *aec7*, *aec8*
Toxin	Hemolysin/cytolysin A	*hlyE/clyA*

**Table 4:** Antibiotic susceptibility testing results.

Classification of Antibiotics	Antibiotic Name	Inhibition Zone Diameter ^a^ (mm)	Antibiotic Resistance
Penicillins	Ampicillin	10	R
	Amoxicillin	8	R
Carbapenems	Meropenem	14	R
	Imipenem	30	S
Cephems	Cefoxintin	19	S
	Ceftriaxone	7	R
	Ceftazidime	10	R
	Cefuroxime sodium	7	R
Tetracyclines	Tetracycline	7	R
	Doxycycline	14	I
Quinolones and fluoroquinolones	Ciprofloxacin	7	R
	Norfloxacin	7	R
	Ofloxacin	7	R
	Enrofloxacin	8	R
	Marbofloxacin ^b^	7	/
Aminoglycosides	Gentamicin	7	R
	Tobramycin	7	R
	Amikacin	7	R
	Kanamycin ^b^	7	/
Chloramphenicols	Florfenicol ^b^	7	/
Sulfonamides	Sulfamethoxazole/trimethoprim	7	R
Polymyxins ^a^	Polymyxin B ^b^	15	/
Macrolides ^a^	Azithromycin ^b^	7	/

^a^ The diameter of the antimicrobial susceptibility disk is 7 mm. ^b^ CLSI does not provide a breakpoint standard for the disk diffusion method.

## Data Availability

The sequence information of the sequencing samples used in the study, including the host, collection site, and date information, has been submitted to GenBank, and the specific registration number information is shown in this article.

## Supplementary Materials

The following supporting information can be downloaded at https://www.mdpi.com/article/10.3390/vetsci12090793/s1, Figure S1: PCR results for viral detection in deceased ostrich 1; Figure S2: PCR results for viral detection in deceased ostrich 2; Figure S3: Linearized comparison between HZDC01 and JL22.
