# Supplementary material for: Antimicrobial Resistance and Genomic Characterization of an Escherichia coli Strain Harboring p0111 and an IncX1-Type Plasmid, Isolated from the Brain of an Ostrich
Source: Vet Sci. 2025 Aug 22;12(9):793. doi: 10.3390/vetsci12090793 (PMC12474207; doi:10.3390/vetsci12090793)
Supplement: Supplementary file 1 [file vetsci-12-00793-s001.zip › vetsci-3789188-supplementary.pdf]

veterinary sciences

Supplementary Materials

Antimicrobial Resistance and Genomic Characterization of an *Escherichia coli* Strain Harboring p0111 and an IncX1-Type Plasmid, Isolated from the Brain of an Ostrich

Jing Hu [1,2,3,†], Jiahe Zhou [4,†], Leping Wang [2,3], Zhongwei Chen [2,3], Yizhou Tan [1,2,3], Yangyan Yin [1,2,3], Zhe Pei [5], Changting Li [2,3], Huili Bai [1,2,3], Chunxia Ma [2,3], Ling Teng [1,2,3], Yongcui Feng [2,3], Xian Li [6,*], Yingyi Wei [1,*] and Hao Peng [2,3,*]

[1] College of Animal Science and Technology, Guangxi University, Nanning 530004, China
[2] Guangxi Key Laboratory of Veterinary Biotechnology, Guangxi Veterinary Research Institute, Nanning 530001, China
[3] Key Laboratory of China (Guangxi)-Association of Southeast Asian Nations (ASEAN) Cross-Border Animal Disease Prevention and Control, Ministry of Agriculture and Rural Affairs of China, Nanning 530001, China
[4] School of the Integrated Chinese and Western Medicine, Hunan University of Chinese Medicine, Changsha 410208, China
[5] School of Neuroscience, Virginia Tech, Blacksburg, VA 24061, USA
[6] Guangxi Agricultural Engineering Vocational Technical College, Nanning 532100, China
* Correspondence: 13788428839@163.com (X.L.); weiyingyi@gxu.edu.cn (Y.W.); hpeng2006@163.com (H.P.)
† These authors contributed equally to this work and share first authorship.

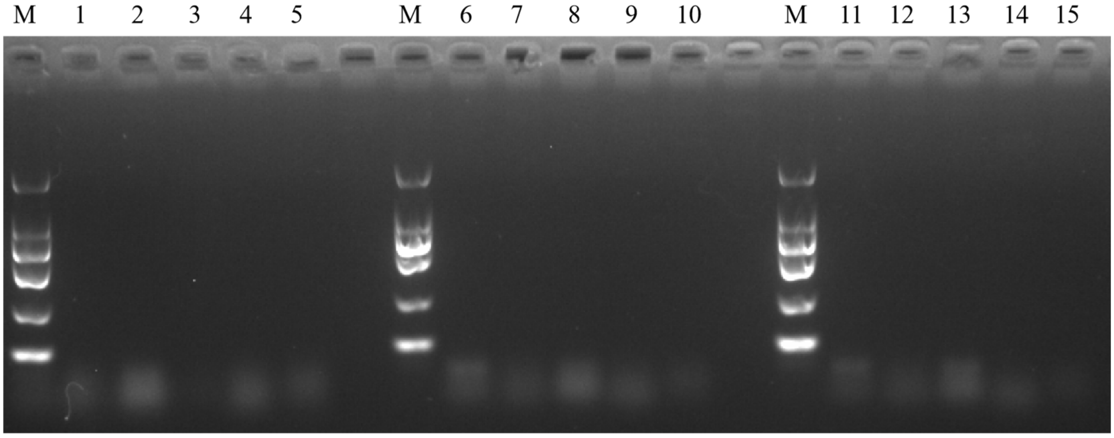

Figure S1. PCR Results for Viral Detection in Deceased Ostrich 1
M: DL 2,000 ladder.
Lane 1 - 5: The intestinal sample was tested for APMV-1, AEV, ARV, GPV, and MS, respectively.
Lane 6 - 10: The liver sample was tested for APMV-1, AEV, ARV, GPV, and MS, respectively.
Lane 11 - 15: The brain sample was tested for APMV-1, AEV, ARV, GPV, and MS, respectively.

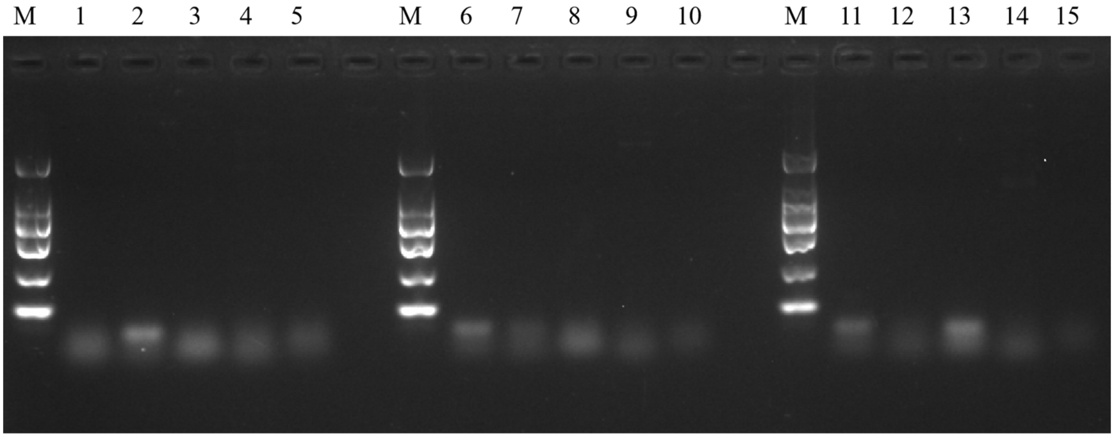

Figure S2. PCR Results for Viral Detection in Deceased Ostrich 2
M: DL 2,000 ladder.
Lane 1 - 5: The intestinal sample was tested for APMV-1, AEV, ARV, GPV, and MS, respectively.
Lane 6 - 10: The liver sample was tested for APMV-1, AEV, ARV, GPV, and MS, respectively.
Lane 11 - 15: The brain sample was tested for APMV-1, AEV, ARV, GPV, and MS, respectively.

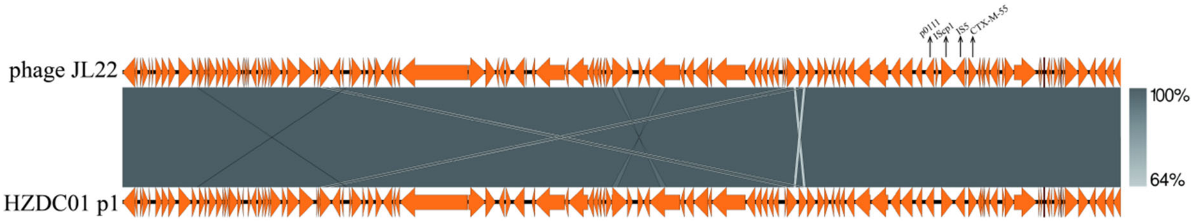

Figure S3. Linearized comparison between HZDC01 and JL22.
